# Supplementary material for: Arsenic Induces M2 Macrophage Polarization and Shifts M1/M2 Cytokine Production via Mitophagy
Source: Int J Mol Sci. 2022 Nov 10;23(22):13879. doi: 10.3390/ijms232213879 (PMC9693596; doi:10.3390/ijms232213879)
Supplement: Supplementary file 1 [file ijms-23-13879-s001.zip › ijms-2016291-supplementary.pdf]

Supplementary Table S1. Mouse blood CD80/CD206 percentages after drinking 50 mg/L of arsenic-containing water for 24 weeks.

Mouse Blood Cells (%)	Control	Arsenic Treated
CD80+	2.3 ± 0.8	2.1 ± 0.7
CD206+	4.0 ± 1.1	* 9.6 ± 1.8

* $p < 0.05$, arsenic treated vs un-treated by the Kruskal-Wallis test (N = 6)

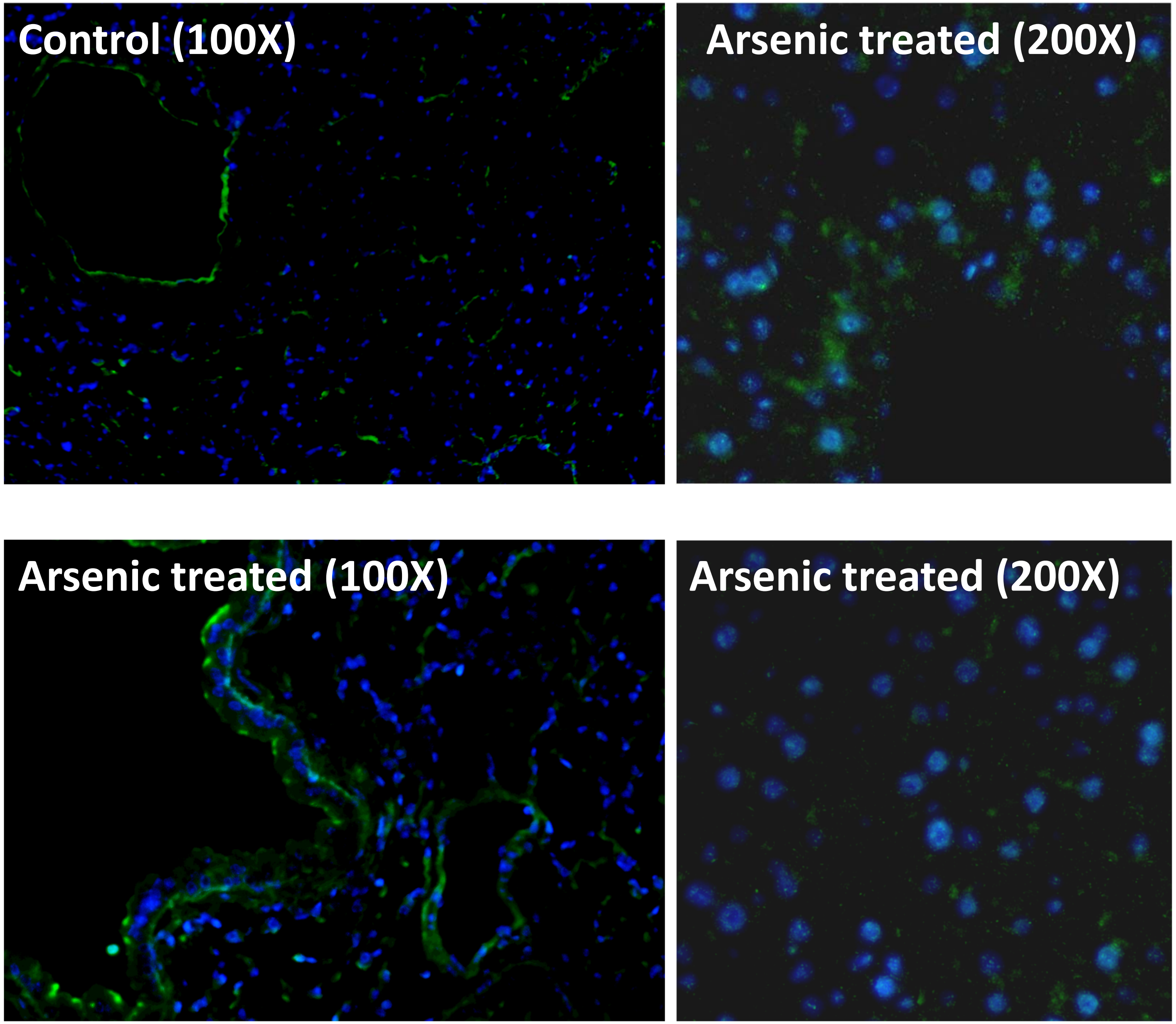

Supplementary Figure S1. Lung tissues form mouse (drinking 50 mg/L of arsenic-containing water for 24 weeks) were stained with CD206 (green) and DAPI. Left: air way view (100x); Right: infiltrated cell view (200x). N=6
